# Association of Batai Virus Infection and Encephalitis in Harbor Seals, Germany, 2016

**DOI:** 10.3201/eid2409.171829

**Published:** 2018-09

**Authors:** Wendy K. Jo, Vanessa M. Pfankuche, Annika Lehmbecker, Byron Martina, Ana Rubio-Garcia, Stefanie Becker, Jochen Kruppa, Klaus Jung, Daniela Klotz, Julia Metzger, Martin Ludlow, Wolfgang Baumgärtner, Erhard van der Vries, Albert Osterhaus

**Affiliations:** University of Veterinary Medicine Hannover, Hannover, Germany (W.K. Jo, V.M. Pfankuche, A. Lehmbecker, S. Becker, J. Kruppa, K. Jung, D. Klotz, J. Metzger, M. Ludlow, W. Baumgärtner, E. van der Vries, A. Osterhaus);; Center for Systems Neuroscience, Hannover (W.K. Jo, V.M. Pfankuche, W. Baumgärtner, A. Osterhaus);; Artemis One Health, Delft, the Netherlands (B. Martina, A. Osterhaus);; Seal Centre, Pieterburen, the Netherlands (A. Rubio-Garcia)

**Keywords:** Batai virus, viruses, Bunyamwera virus, Ngari virus, orthobunyavirus, infection, encephalitis, meningitis/encephalitis, harbor seals, Phoca vitulina, zoonoses, Germany

## Abstract

We isolated Batai virus from the brain of a euthanized, 26-year-old, captive harbor seal with meningoencephalomyelitis in Germany. We provide evidence that this orthobunyavirus can naturally infect the central nervous system of a mammal. The full-genome sequence showed differences from a previously reported virus isolate from a mosquito in Germany.

Batai virus (BATV) is a member of the Bunyamwera serogroup of orthobunyaviruses of the family *Peribunyaviridae*. Orthobunyaviruses are single-stranded, negative-sense RNA viruses with a tripartite genome composed of small, medium, and large segments, which encode nucleocapsid, glycoproteins, and polymerase, respectively (*1*). These segments can be interchanged between viruses of the same genus, resulting in stable reassortant bunyaviruses. For example, the Ngari virus genome consists of segments from BATV and Bunyamwera virus. Ngari virus is associated with outbreaks of hemorrhagic fever in humans and shows a clinical spectrum different from that of both parent viruses (*2*).

BATV has been documented to cause mild illness in ruminants and humans (*3*,*4*). Other hosts include domestic pigs and wild birds (*3*). BATV is transmitted mainly by *Anopheles* and *Culex* spp. mosquitoes and is widely distributed throughout Europe, Asia, and Africa (*3*).

In Germany, BATV was first detected in *Anopheles maculipennis* mosquitoes in 2009 (*5*). Enzootic transmission cycles involving domestic and wild mammals was reported in a serologic study in which 3 (0.55%) of 548 cattle had BATV-neutralizing serum antibodies (*6*). We report natural BATV infection of 2 captive harbor seals (*Phoca vitulina*), in Germany, in which meningoencephalomyelitis developed in 1 of them.

## The Study

**Figure 1:**
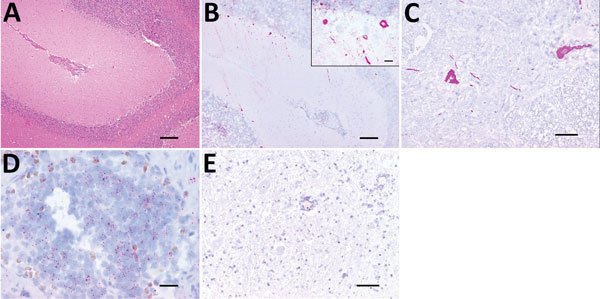
Histologic analysis and fluorescent in situ hybridization (FISH) of a Batai virus BATV)–infected harbor seal, Germany, 2016. A) Cerebellum showing mild to moderate, perivascularly accentuated, lymphohistiocytic inflammation (hematoxylin and eosin stain; scale bar indicates 200 μm). B) Purkinje cells and neurons of granular cell layer showing intracytoplasmic BATV–specific pink, positive result detected by FISH (fast red stain; scale bar indicates 200 µm). Inset: Higher magnification view of analysis using the QuantiGene ViewRNA ISH Tissue 1-Plex Assay Kit and the QuantiGene ViewRNA Chromogenic Signal Amplification Kit (Affymetrix-Panomics, Santa Clara, CA, USA) (fast red stain; scale bar indicates 20 µm). C) Scattered neurons of spinal cord showing a strong, pink, intracytoplasmic BATV-specific result detected by FISH (fast red stain; scale bar indicates 100 µm). D) Cortical and medullary lymphocytes of pulmonary lymph node showing a mild, pink, intracytoplasmic BATV-specific result detected by FISH (fast red stain; scale bar indicates 20 µm). E). Negative control (incubation without probe) of spinal cord showing no BATV-specific result (fast red stain; scale bar indicates 100 µm).

In September 2016, a 26-year-old male harbor seal (*Phoca vitulina*) in a zoo in northern Germany showed peracute deterioration of its general condition. Because of progression and severity of illness, the seal was euthanized and the carcass sent to the Department of Pathology, University of Veterinary Medicine Hannover (Hannover, Germany), for necroscopic analysis. Macroscopic examination showed signs of distress but no gross lesions were detected. Histologic analysis showed a mild to moderate, multifocal, perivascularly accentuated, lymphohistiocytic meningoencephalomyelitis, which affected the cerebrum, cerebellum (Figure 1, panel A), brain stem, medulla oblongata, and cervical spinal cord. Histologic analysis indicated a virus etiology.

Routine immunohistochemical tests of the seal brain for morbilliviruses, Borna disease virus, and tick-borne encephalitis virus (*7*,*8*) and immunofluorescence analysis for rabies virus were performed by the Department of Consumer and Food Safety of Lower Saxony (Hannover, Germany). All tests showed negative results.

We attempted virus isolation from homogenized brain in Vero cells. Cytopathic changes were observed within 3 days and continued to emerge in subsequent passages. To identify the pathogen, we investigated supernatant from the initial Vero cell isolation by using deep sequencing and a modified sequence-independent, single-primer amplification protocol as described (*9*,*10*). Analysis of raw reads with Bowtie 2 version 2.2.9 (https://sourceforge.net/projects/bowtie-bio/files/bowtie2/2.2.9/) for DNA mapping and Pauda version 1.0.1 (https://bioconda.github.io/recipes/pauda/README.html) for amino acid mapping identified BATV.

**Figure 2:**
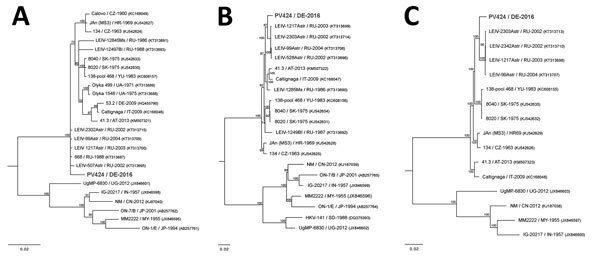
Bayesian phylogeny trees based on full-genome coding region sequences of small, medium, and large RNA segments of Batai virus and comparison viruses. A) Small RNA segments (69–770 bp). Bunyamwera virus (GenBank accession no. D00353) was used as the outgroup. B) Medium RNA segments (42–4,346 bp). Bunyamwera virus (GenBank accession no. M11852) was used as the outgroup. C) Large RNA segments (49–6,762 bp). Bunyamwera virus (GenBank accession no. X14383) was used as the outgroup. Bold indicates virus isolated in this study. Analysis was performed for 1 million generations and sampled every 100 steps. The first 25% of samples were discarded as burn-in according to MrBayes (*11*). Hasegawa-Kishino-Yano nucleotide substitution model was selected as best-fit model according to Bayesian information criteria. Numbers at the nodes indicate posterior probabilities percentage. GenBank accession numbers are provided for comparison isolates; accession nos. of the isolated Batai virus strain PV424/DE-2016 are small, MH299972; medium, MH299973; large, MH299974. Scale bars indicate nucleotide substitutions per site.

We created a reference assembly for all 3 genome segments (GenBank accession nos. S, MH299972; M, MH299973; and L, MH299974) by using CLC Genomics Workbench version 9.0 (QIAGEN, Hilden, Germany). The isolated virus was closely related to previously identified BATV strains from Europe (Figure 2) but had the highest sequence homology with strains from Russia (nucleotide pairwise identity S, 99%; M, 98.6%; and L, 98.5%).

We tested seal tissues (Table) for BATV by using real-time PCR and fluorescent in situ hybridization (FISH) as described (*5*,*12*). We used BATV-specific probe 5′-FAM-aacagtccagttccagacgatggtc-BHQ-1-3′ and primers Fwd-5′-Gctggaaggttactgtatttaatac-3′ and Rv-5′-Caaggaatccactgagtctgtg-3′ specific for the S segment (*5*). A BATV-specific probe for nucleotides 28–899 of the S segment was designed for FISH experiments (QuantiGene ViewRNA Kits; Affymetrix-Panomics, Santa Clara, CA, USA), which were performed according to the manufacturer’s protocol with minor modifications (*12*).

**Table:** Analysis of a Batai virus–infected harbor seal with meningoencephalomyelitis, Germany, 2016*

Sample material	Histopathologic finding	Real-time PCR (cycle threshold)†	FISH
Brain	Cerebrum, cerebellum, brain stem, medulla oblongata, and cervical spinal cord: mild to moderate, multifocal, and lymphohistiocytic meningoencephalomyelitis, perivascularly accentuated; parietal lobe: multiple glial nodules; thoracic spinal cord: mild to moderate and multifocal meningitis, perivascularly accentuated, lymphohistiocytic with few eosinophilic granulocytes; cauda equina: mild to moderate, multifocal, and lymphohistiocytic perineuritis	+ (15)	+
Lung	Mild and multifocal anthracosis; acute, diffuse, and severe hyperemia; acute, diffuse, and moderate edema	– (>35)	–
Spleen	Moderate to severe and diffuse hyperemia	– (35)	–
Kidney	Mild, interstitial, and lymphohistiocytic nephritis with single, intratubular concrements	– (>35)	–
Pulmonary lymph node	Mild follicular hyperplasia	NI	­+
Mesenteric lymph node	Mild to moderate follicular hyperplasia and hemosiderosis	NI	–
Liver	Mild, multifocal, lymphohistiocytichepatitis, mild to moderate hepatocellular storage of iron	– (>35)	–
Small intestine	Mild, diffuse, lymphoplasmacytic, and partially eosinophilic enteritis	+ (28)	+
Large intestine	NSML	NI	–
Nose	NSML	NI	–
Heart	NSML	NI	–
Stomach	NSML	NI	–

*FISH, fluorescent in situ hybridization; NI, not investigated; NSML, no major microscopic lesions; –, negative; +, positive. †Negative result >35; positive result <35.

The highest virus load (by real-time PCR) was found in the central nervous system, and lesion-associated Purkinje cells and neurons of the granular cell layer of the cerebellum showed positive FISH results (Figure 1, panel B). A positive cytoplasmic result was also obtained for single spinal cord neurons (Figure 1, panel C). More limited BATV infection was found in peripheral organs, and the lowest cycle threshold was for the intestine. We also found BATV in single cells of the tunica mucosa of the small intestine and in cortical and medullary lymphocytes of the pulmonary lymph node by FISH (Figure 1, panel D). Other organs showed negative results in both assays.

We also performed histopathologic analysis of archived formalin-fixed paraffin-embedded (FFPE) organ samples of a seal that had shared the enclosure with the BATV-infected seal and had died 2 months before the euthanized seal showed the first clinical signs. Glomerular and tubular epithelial kidney cells (Technical Appendix Figure), cells of the tunica mucosa of the small intestine, and cortical and medullary lymphocytes of the pulmonary lymph node showed positive results for BATV by FISH. These FFPE samples did not show positive results by real-time PCR, probably because of low sensitivity of the assay for FFPE samples (*13*).

Retrospective analysis of FFPE brain samples of seals (n = 7) that had histopathologic changes suggestive of an unknown virus etiology and were isolated from harbor seals in coastal waters of Germany in the past decade all had negative results for BATV by FISH. Therefore, we screened 100 serum samples from harbor seals and 100 serum samples from gray seals (*Halichoerus grypus*) collected in 2016 and 2017 after admission to a seal rehabilitation center in the Netherlands that covers seal populations partially overlapping those of coastal waters of Germany. We neutralized isolated seal BATV (100 50% tissue culture infective doses) with diluted serum samples before application to reporter cells and examined for cytopathic effects after 3 days. However, no BATV antibodies (titer >1:20) were detected.

## Conclusions

We isolated and characterized BATV from the brain of a captive harbor seal in Germany. This seal had lymphohistiocytic meningoencephalomyelitis and evidence of virus replication in Purkinje cells, neurons, enterocytes, and lymphocytes in peripheral tissues. Evidence of BATV infection by FISH was also obtained for a second harbor seal that had died 2 months before in the same enclosure. No additional evidence was found for seals as natural hosts for BATV infection by investigating brains from seals with encephalitis in coastal waters of Germany and by conducting a serosurvey among free-living harbor and gray seals. Results obtained from the 2 BATV infected animals indicated BATV circulation in the area during the mosquito season and that captive seals were possible dead-end hosts. Because seals in their natural environment are most likely less exposed to mosquitoes than seals in captivity, the observed seal BATV infections might be unnatural captivity-associated events. Phylogenetic analysis indicated that BATV isolated from the seal brain differed from BATV isolated from a mosquito in Germany and is more closely related to strains identified in Russia.

This study provides evidence of BATV associated with central nervous system disease in a naturally infected mammal. Other orthobunyaviruses have also been shown to cross the blood–brain barrier and show neurotropic properties (*14*). For example, a virus from the same serogroup, Bunyamwera virus, was recently associated with neurologic disease and abortion in horses (*15*). Moreover, possible human BATV infection in disease-endemic regions requires further investigation because BATV infection of mammals, including humans, has been reported in Europe (*3*) and Sudan (*4*).

Furthermore, BATV is the donor of the M segment of Ngari virus, which causes hemorrhagic fever in humans (*2*). The 2 BATV-infected seals could have been exceptionally sensitive to BATV infection because of predisposing factors, such as advanced age, concurrent conditions, genetic predisposition, or immunologic deficiencies. This possibility raises the question whether immunocompromised humans or other mammals might be at increased risk for development of neurologic BATV infection. Collectively, our data indicate the need for increased surveillance of BATV infection in mosquitoes, mammals, and birds in Europe.

Technical Appendix Additional information on Batai virus infection and encephalitis in harbor seals, Germany, 2016.
