# Supplementary material for: Association of Batai Virus Infection and Encephalitis in Harbor Seals, Germany, 2016
Source: Emerg Infect Dis. 2018 Sep;24(9):1691–5. doi: 10.3201/eid2409.171829 (PMC6106443; doi:10.3201/eid2409.171829)
Supplement: Technical Appendix — Additional information on Batai virus infection and encephalitis in harbor seals, Germany, 2016. [file 17-1829-Techapp-s1.pdf]

Article DOI: https://doi.org/10.3201/eid2409.171829

Association of Batai Virus Infection and Encephalitis in Harbor Seals, Germany, 2016

Technical Appendix

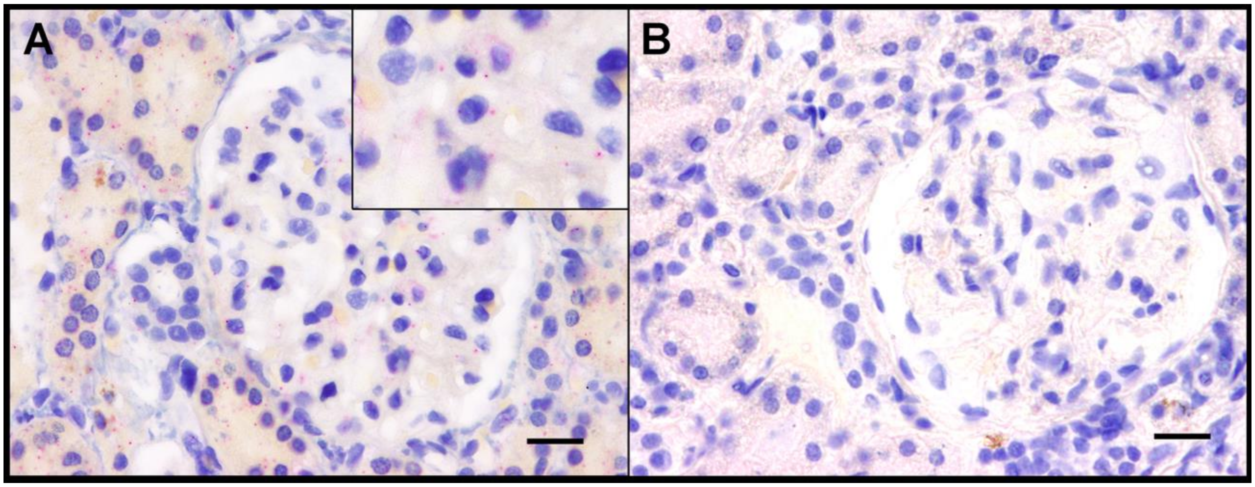

Technical Appendix Figure. Fluorescent in situ hybridization (FISH) of kidney cells from harbor seal infected with Batai virus, Germany, 2016. A) Cells within glomeruli and tubular epithelial cells showing a mild pink, intracytoplasmic BATV-specific result detected by FISH. Inset: Higher magnification view (fast red stain). B) Kidney tissue used as negative control (incubation without probe). Scale bars indicate 20 µm.
